# The Potential Role of Previous Physical Exercise Program to Reduce Seizure Susceptibility: A Systematic Review and Meta-Analysis of Animal Studies

**DOI:** 10.3389/fneur.2021.771123

**Published:** 2021-12-10

**Authors:** Ricardo Mario Arida, Adrielle Andrade Passos, Alexandre Lebedev Graciani, João Angelo Ferres Brogin, Mayara de Almeida Lima Ribeiro, Jean Faber, Robson Campos Gutierre, Lavinia Teixeira-Machado

**Affiliations:** ^1^Department of Physiology, Federal University of São Paulo, São Paulo, Brazil; ^2^Department of Education in Health, Federal University of Sergipe, São Cristóvão, Brazil; ^3^Department of Mechanical Engineering, São Paulo State University, São Paulo, Brazil; ^4^Department of Neurology and Neurosurgery, Federal University of São Paulo (UNIFESP), São Paulo, Brazil

**Keywords:** seizure susceptibility, physical exercise, epilepsy, animal model, brain resilience

## Abstract

**Background:** Clinical and pre-clinical studies indicate a reduction in seizure frequency as well as a decrease in susceptibility to subsequently evoked seizures after physical exercise programs. In contrast to the influence of exercise after epilepsy previously established, various studies have been conducted attempting to investigate whether physical activity reduces brain susceptibility to seizures or prevents epilepsy. We report a systematic review and meta-analysis of different animal models that addressed the impact of previous physical exercise programs to reduce seizure susceptibility.

**Methods:** We included animal model (rats and mice) studies before brain insult that reported physical exercise programs compared with other interventions (sham, control, or naïve). We excluded studies that investigated animal models after brain insult, associated with supplement nutrition or drugs, that did not address epilepsy or seizure susceptibility, *ex vivo* studies, *in vitro* studies, studies in humans, or *in silico* studies. Electronic searches were performed in the MEDLINE (PubMed), Web of Science (WOS), Scopus, PsycINFO, Scientific Electronic Library Online (SciELO) databases, and gray literature, without restrictions to the year or language of publication. We used SYRCLE's risk of bias tool and CAMARADES checklist for study quality. We performed a synthesis of results for different types of exercise and susceptibility to seizures by random-effects meta-analysis.

**Results:** Fifteen studies were included in the final analysis (543 animals), 13 of them used male animals, and Wistar rats were the most commonly studied species used in the studies (355 animals). The chemoconvulsants used in the selected studies were pentylenetetrazol, penicillin, kainic acid, pilocarpine, and homocysteine. We assessed the impact of study design characteristics and the reporting of mitigations to reduce the risk of bias. We calculated a standardized mean difference effect size for each comparison and performed a random-effects meta-analysis. The meta-analysis included behavioral analysis (latency to seizure onset, *n* = 6 and intensity of motor signals, *n* = 3) and electrophysiological analysis (spikes/min, *n* = 4, and amplitude, *n* = 6). The overall effect size observed in physical exercise compared to controls for latency to seizure onset was −130.98 [95% CI: −203.47, −58.49] (seconds) and the intensity of motor signals was −0.40 [95% CI: −1.19, 0.40] (on a scale from 0 to 5). The largest effects were observed in electrophysiological analysis for spikes/min with −26.96 [95% CI: −39.56, −14.36], and for spike amplitude (μV) with −282.64 [95% CI: −466.81, −98.47].

**Discussion:** *Limitations of evidence*. A higher number of animal models should be employed for analyzing the influence of exerciseon seizure susceptibility. The high heterogeneity in our meta-analysis is attributable to various factors, including the number of animals used in each study and the limited number of similar studies. *Interpretation*. Studies selected in this systematic review and meta-analysis suggest that previous physical exercise programs can reduce some of the main features related to seizure susceptibility [latency seizure onset, spikes/min, and spike amplitude (μV)] induced by the administration of different chemoconvulsants.

**Systematic Review Registration:** PROSPERO, identifier CRD42021251949; https://www.crd.york.ac.uk/prospero/display_record.php?RecordID=251949.

## Introduction

Epilepsy is a common neurological disorder that affects over 70 million people worldwide and it is characterized by an enduring predisposition to generate spontaneous epileptic seizures, resulting in neurobiological, cognitive, psychological, and social consequences (1). The neurophysiological basis of epileptic seizures has been associated with an imbalance between neuronal excitatory and inhibitory activity in the brain (2, 3). The most common treatments for epilepsy include anti-epileptic drugs (AEDs), epilepsy surgery, and device therapies such as *vagus* nerve stimulation, deep brain stimulation, and a ketogenic diet. Apart from the conventional treatments for epilepsy, alternative neuroprotective and antiepileptogenic approaches have been used for the prevention and treatment of epilepsy. Among them, physical exercise has been successfully cited as an adjunctive form of treatment for epilepsy (4).

Clinical studies have consistently demonstrated that regular exercise positively impacts epilepsy (5–8). Investigations primarily focusing on exercise-induced effects on seizure frequency have shown improvement in seizure control (5, 8) or no increase in seizure frequency after physical exercise programs (9, 10). In addition, studies that analyzed whether intensive exercise could interfere in seizure susceptibility have not reported seizures during the incremental effort to exhaustion in an ergometric test or after physical exertion (11–13). In addition to the positive findings reported in epilepsy conditions, regular exercise over the course of life has been associated with resilience to developing epilepsy. The few human studies that have explored this issue have shown that regular physical exercise was associated with a low incidence of epilepsy. For instance, low cardiovascular fitness at the age of 18 years was associated with an increased risk of epilepsy later in life (14). Moreover, the incidence of epilepsy analyzed over a period of 20 years was lower in high-fit subjects (cross-country ski racers) before retirement compared with the incidence of their non-active controls (15).

Although the beneficial influence of exercise in humans has been reported in literature, the mechanisms by which exercise positively impacts on epilepsy are yet to be elucidated. Animal models of seizures and epilepsy are valuable tools for understanding the pathophysiology of the disease and in developing new treatments (16). They have contributed significantly to identifying the mechanisms of cellular hyperexcitability, alterations triggering the transition from an interictal to an ictal state, seizure propagation and termination, mechanisms and side effects of antiepileptic drugs, as well as behavioral manifestations caused by seizures (16). In this regard, an increasing number of animal studies have demonstrated the impact of exercise in epilepsy and the neurobiological mechanisms of these beneficial effects (4, 17). Two systematic reviews and meta-analyses addressed the efficacy of exercise before and after the induction of epilepsy, using the pilocarpine and kainate models of epilepsy (18, 19). In their review, the physical exercise program significantly reduced the number of spontaneous recurrent seizures in the pilocarpine and kainate models (18, 19). Physical exercise also exerted a positive influence before pilocarpine and kainate induction by increasing the latency to develop *status epilepticus* (SE), although not statistically significant in the pilocarpine model. Indeed, some discrepancies still exist in the results of studies evaluating the potential effect of exercise to prevent the development of epilepsy.

Under this context, to better establish whether regular physical exercise is effective in reducing seizure susceptibility and epilepsy development, extensive analysis with the inclusion of different models of seizures and epilepsy is needed to draw better conclusions. Therefore, we conducted a systematic review and meta-analysis of studies in animal models of seizure/epilepsy to determine the extent to which different types of physical exercise influences seizure susceptibility and epilepsy development.

## Methods

### Review Protocol

This systematic review and meta-analysis were compliant with the Preferred Reporting Items for Systematic Reviews and Meta-analysis (PRISMA) statement (20, 21). The PRISMA guideline is commonly used for clinical trials, but we adapted it for this systematic review (22). The protocol was based on SYRCLE's systematic review protocol format for animal intervention studies (23).

### Eligibility Criteria

We considered behavioral and electrophysiological analysis induced by exercise before brain insult outcomes measures for synthesis analysis, according to the following inclusion and exclusion criteria:

Inclusion Criteria: studies that addressed physical exercise programs before brain insult in animal models (rats and mice) using treadmills, voluntary wheel running, swimming, inclined vertical ladder apparatus, progressive resistance wheel exercise, and compared with other interventions (sham, control, or naïve). Exclusion criteria: studies that investigated animal models after brain insult, associated with supplement nutrition or drugs, or that did not address epilepsy or seizure susceptibility. We excluded *ex vivo* studies, *in vitro* studies, studies in humans, or *in silico* studies.

### Information Sources

The electronic search strategy of this review was performed to identify studies about physical exercise programs to identify seizure susceptibility on animal models until June 2021. Specific strategy in databases [MEDLINE (PubMed, we included Syrcle animal filter), Web of Science (WOS), Scopus, PsycINFO, and Scientific Electronic Library Online (SciELO)] were: topic: (“exercise”[MeSH Terms] OR “physical exercise”[All Fields] OR “exercise program”[All Fields] OR “physical program”[All Fields]) AND (“epilepsy”[MeSH Terms] OR “seizure”[MeSH Terms]); ALL (“exercise”) AND TITLE-ABS-KEY (“Physical program” OR “Physical exercise”) AND TITLE-ABS-KEY (“epilepsy”) OR (“seizure”) AND TITLE-ABS-KEY (animal OR animals OR rat OR rats OR rodents OR mice OR mouse OR murine). Freehand searching, gray literature, reference lists, the world wide web, and personal collections of articles were utilized by the authors using search terms and boolean operators [(exercise program) AND (epilepsy)] were included.

### Search Strategy

A systematic literature search was conducted to identify studies on physical exercise programs before brain insult that addressed seizure susceptibility in animal models. This systematic review composed the question research based on PICOS: P—population, I—intervention, C—comparative interventions, O–outcomes, S–type of study. For the question “What is the influence of physical exercise programs on seizure susceptibility in all experimental animal models?,” the PICOS was: P—animal models (rats, mice, gerbils); I–physical exercise; C—controls, naïve, or sham; O—seizure susceptibility; and S—experimental studies. We included studies investigating the relationship between physical exercise programs and susceptibility to seizures in animal models until June 2021 (21). The complete search strategy can be accessed in supplementary content. The search strategy included epilepsy, seizure susceptibility, physical exercise, and animal model. The electronic search was performed in the MEDLINE (PubMed), Web of Science (WOS), Scopus, PsycINFO, and Scientific Electronic Library Online (SciELO) databases, without restrictions in the year or language of publication.

### Selection Process

Two reviewers (AAP and MALR) on the stages of the review protocol independently selected the titles and abstracts to ensure that the studies met the predefined eligibility criteria. To avoid discrepancy between reviewers, the following exclusion criteria were (1) non-animal model; (2) studies that investigated animal models after brain insults; (3) acute exercise protocol; (4) exercise associated with nutritional supplements or medications; (5) measures that did not address the susceptibility to seizures. Studies considered unrelated to the research issue and study reviews were excluded at this stage. The two reviewers accessed and selected the other full articles and considered the eligibility criteria. Disagreements and discrepancies were resolved by consensus after discussion with two other reviewers (LTM & RMA). The inter-rater reliability was evaluated by the Kappa test where values ≤ 0 as indicating no agreement and 0.01–0.20 as none to slight, 0.21–0.40 as fair, 0.41–0.60 as moderate, 0.61–0.80 as substantial, and 0.81–1.00 as almost perfect (24).

### Data Collection Process

We used Review Manager (RevMan, version 5.4) for data extraction. The data were extracted from text, tables, and graphs. The main outcome is the influence of physical exercise programs on seizure susceptibility. According to study design, we created a form to extract data related to study design (exercise vs. control, sham, or naive), number of experimental groups, number of animals per group, comparison conditions, duration, frequency, time points measured, outcome measures, and primary and secondary outcomes. According to the animal models, information about sample descriptions (animal, sex, age, weight, etc), behavior (seizures susceptibility measures), morphological, electrophysiological, and molecular analysis induced by exercise before brain insult in rats and/or mice, both male and female. For the intervention of interest, we extracted studies that addressed the effects of physical exercise programs on seizure susceptibility of rodents, with two or more experimental groups, and that present comparison conditions (control and/or sham). We extracted duration, frequency, time points measured data, outcome measures, primary and secondary outcomes. The primary outcome was the influence of exercise on seizure susceptibility by dichotomous data. This variable was extracted by the following measures: behavioral manifestations of (a) latency of the first motor signs (seconds), (b) latency for reaching a seizure or *status epilepticus* (seconds), (c) number of animals that developed seizures or *status epilepticus*, (d) intensity of motor symptoms, (e) mortality and electrophysiological analysis [spike frequency (spike/min) and amplitude (μV)] (median 25–75% or mean ± SD). These continuous data were extracted according to the information presented in selected studies.

### Study Risk of Bias and Quality of the Selected Studies Assessments

We used the SYRCLE risk of bias (RoB) tool and the CAMARADES checklist for the quality of the studies (23). Two reviewers (AAP and ALG) analyzed the risk of bias and evaluated the quality of the studies, minimizing variance. The discrepancies were resolved by consensus with the third reviewer (RMA). Two reviewers (AAP and ALG) determined the quality of evidence for each result and how it can be applied in specific interventions and populations. The color “green” was used to indicate that the criterion was declared, and implied a low risk of bias, “yellow” was used to indicate that the criterion was not declared and implied a high risk of bias, and “red” was used to indicate an unclear statement and implied a high risk of bias. For the purpose of this review, we considered studies of superior quality if they reported a low risk of bias in at least half of the classification criteria.

### Meta-Analysis

Meta-analyses were performed for the different types of exercise, considering four main features associated with seizure susceptibility: latency to seizure onset, the intensity of motor signals, spike rate, and spike amplitude.

As discussed in Tufanaru et al. (25), under the random-effects model the combined estimation is not an estimate of one fixed value, but rather the average of a distribution of values. It is mainly adopted when it is not possible to assume there is only one true effect size which is shared by all the included studies and, hence, the true effect varies from study to study. In this work, since it is of great interest to generalize the possible benefit of exercises in mitigating the effects of epileptic seizures, but the animal models in the studies selected are different (and thus it cannot be assumed that they come from the same distribution), a random-effects model is applied. Although the number of studies selected is not high, the use of the random-effects model distributes the weights of each study according to the between-studies variance, preserving the statistical information from studies with a low number of samples.

Essentially, if one seeks to assess the overall effect of *n* studies using intervention (1) and control (2) groups, this corresponds to calculating *MD*_*n*_ mean differences and weighted standard deviations *s*_*n*_ to generate *n* confidence intervals, respectively, given by Borenstein et al. (26):


(1) $$\begin{array}{r} MD_{n}={\bar{x}}_{n,1}-{\bar{x}}_{n,2} \end{array}$$

(2) $$\begin{array}{r} s_{n}=\sqrt{\frac{s_{n,1}}{N_{n,1}}+\frac{s_{n,2}}{N_{n,2}}} \end{array}$$

(3) $$\begin{array}{r} IC\left(\alpha \right)=\left[MD_{n}-z_{\alpha }s_{n},MD_{n}+z_{\alpha }s_{n}\right] \end{array}$$

where ${\bar{x}}_{n,1}$ and ${\bar{x}}_{n,2}$ are the individual sample means of intervention and control groups for the study, respectively; *N*_*n*, 1_ and *N*_*n*, 2_ are their respective sample sizes, and *z*_α_ is usually 1.96 for α = 0.05. At last, the result is summarized on a single sample, which is pooled according to a pre-defined criterion. In this work, such a criterion is the inverse variance (26):


(4) $$\begin{array}{r} w_{n}=\frac{1}{{s^{2}}_{n}+T^{2}} \end{array}$$

where *w*_*n*_ is the weight associated with each study. In this way, the studies are weighed according to the within-study variance ${s^{2}}_{n}$ and the estimate of the between-studies variance *T*^2^, which, in turn, is calculated by the DerSimonian-Laird method (26).

Another important aspect to be considered is the type of statistical test—left-oriented and right-oriented. If the hypothesis tested implies that the intervention intends to decrease the magnitude of the effect under analysis, $MD_{n}={\bar{x}}_{n,2}-{\bar{x}}_{n,1}$ can be adopted, whose expected results are negative, for example. This issue is addressed and commented on in detail in coming subsections.

We considered controlled experimental studies with different treatment groups that investigated the influence of the exercise program on susceptibility to seizures in animal models. These studies observed behavior (measures of susceptibility to seizures) and electrophysiological analysis before brain insult. The values of the group were adjusted for the standard mean difference according to the outcome quantifications presented in the original articles. For all the results concerning the meta-analysis presented herein, a computational package called *PyMeta* was used (27), implemented in Python 3.7, on the Spyder platform, which is free and open-source.

#### Subgroup Analysis and Investigation of Heterogeneity

We found an link between exercise and non-intervention seizure susceptibility. We stratified and showed separate groups to show effect size differences. The differences between the different types of experiments in the results of the included studies were reduced by the focus of the inclusion and exclusion criteria on the general effect of exercise on seizure susceptibility and variation in the size of the effect in the animal model. Heterogeneity was evaluated by *χ*^2^-statistics followed by *I*^2^ and its confidence interval CI (95%) described according to Borenstein et al. (26). The overall effect was evaluated by the Z-value where significant effects were considered when *p* < 0.05.

#### Sensitivity Analysis

A sensitivity analysis was performed to substitute alternative decisions or ranges of values for arbitrary decisions or unclear, as time-to-event data, continuous data, ordinal scales, dichotomous, or continuous outcomes, as well as following the random-effects method.

#### Synthesis Methods

**Figure 1:**
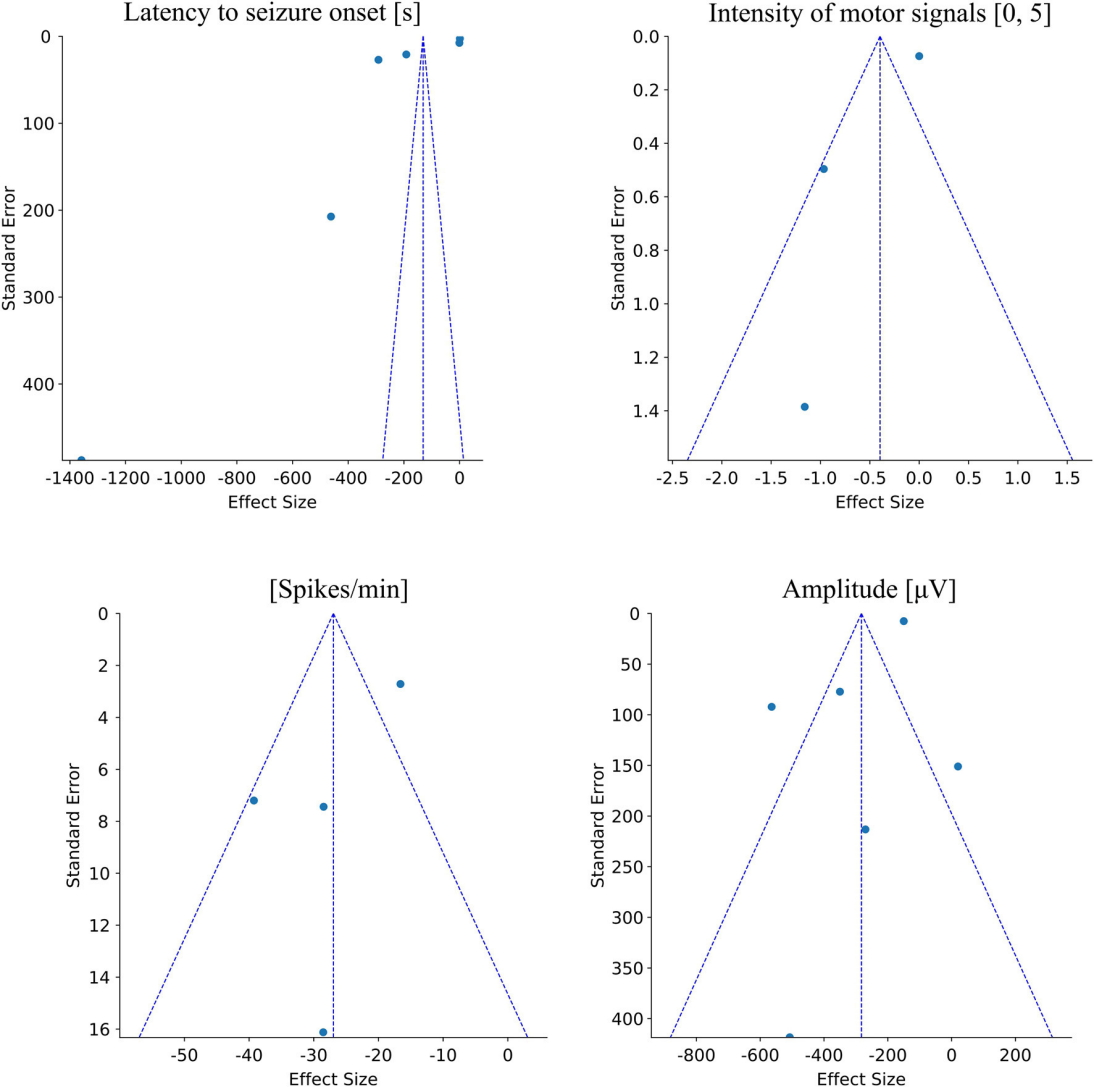
Funnel plots related to each evaluated epileptogenic feature in animal models. The first plot shows the dispersion of the mean effects found in each study related to the latency to seizure onset feature. It is observed that there is an asymmetry among the samples with a leftward trend and high dispersion of the values relative to the limits of the funnel. However, all the effects are located at the top of the funnel showing a low standard error among studies. The second plot shows the dispersion of the effects found in each study related to the intensity of motor signal features. In this plot, it is observed the high heterogeneity among studies and high standard error. Furthermore, since there are only three studies, this plot shows the difficulty in quantitative analysis. The third plot shows the dispersion of the effects found in each study regarding the spikes/min feature. In this plot, it is possible to observe a considerable dispersion among the samples, mainly due to one study showing a high standard error. Although there is a significant overall effect favorable to physical exercise, corroborated by the asymmetry in the funnel plot, this dispersion pattern also demands an increase in the number of studies to improve the stability of the meta-analysis inference. The fourth figure shows the dispersion of the effects found in each study regarding the amplitude of spikes feature. It is possible to observe an effect of dispersion symmetry with a good homogeneity in the standard error among most studies. There is only one sample with a high standard error, but the lateralization of the overall effect in favor of exercise in the mitigation of epileptogenic effects corroborates the meta-analysis.

We reported important findings concerning the influence of physical exercise on seizure susceptibility. In this meta-analysis, we included the primary outcome measure of interest for each comparison, calculation of effect sizes and summary effect sizes, potential sources of heterogeneity, and internal validity. We detected the size of the effect of the selected studies, and whether or not between two effects exercise reduces seizure susceptibility in the animal model, giving a specific factor: latency to seizure onset, the intensity of motor signals, spikes/min, and spike amplitude. The mean difference was used to summarize the same result on the difference in the means of the selected studies. According to the result, the random-effects model was determined as the best average classification effect. Correlated error estimates and/or multiple comparisons may have occurred due to the heterogeneity of the studies. We included funnel plot analysis to display the effects of studies that may be due to reasons other than publication bias, such as poor methodological quality, inadequate analysis, heterogeneity, artifacts (Figure 1).

## Results

### Study Selection

**Figure 2:**
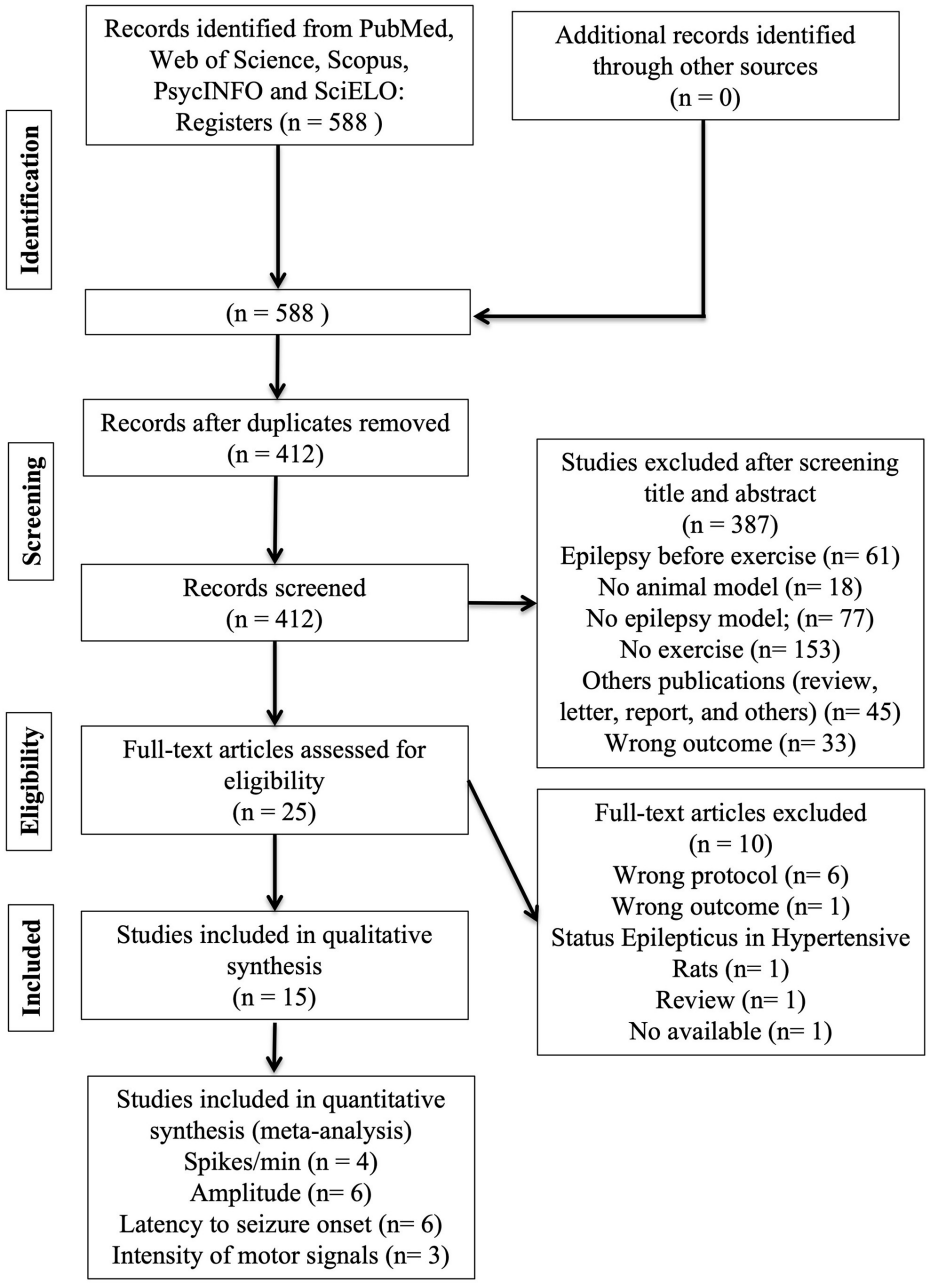
A flow diagram of the systematic review literature search.

The flow of studies is shown in Figure 2. A total of 412 reports were identified in the database search. After screening the titles and abstracts, 25 studies were assessed for eligibility, and 10 were excluded because they did not meet our eligibility criteria, resulting in 15 full-text articles in this systematic review. The inter-rater reliability was evaluated by the Kappa test (0.98 in the first stage and 0.88 in the second stage), indicating agreement between reviewers.

### Included Studies

The 15 studies that met the eligibility criteria included 543 animals, weighing 34–350 g, age 35–150 days, and 13 of them used male animals (see Table 1). Wistar rats were the most common species used in the studies (355), followed by Sprague-Dawley rats (150), ICR mice (40), and Mongolian gerbils (14). Only studies that used exercise intervention before SE induction were included. Six studies used a treadmill (28–33), five studies used swimming (34–38), two studies used a running wheel (39, 40), and two studies used combined exercise: one applied treadmill and swimming (41), and the other used running wheel and treadmill (42). The total duration of exercise ranged between 21 and 90 days. Session duration ranged from 15 to 60 min. The frequency ranged from five to seven times a week, and two studies did not report session frequency and duration (39, 40). Among the chemoconvulsants used in the selected studies, two used pentylenetetrazol (34, 35), five used penicillin (30–33, 36), four used kainic acid (37–40), three pilocarpine (28, 41, 42), and one homocysteine (29). See details in Table 1.

**Table 1:** Summary of included study design, procedures and outcomes.

**Summary of study design, procedures and outcomes**													
**1st Author** **(year)**	**Country**	**Animal**					**Exercise** **regime**	**Total** **duration**	**Frequency-day/week**	**Duration/session (min)**	**Drug** **administration**	**Assessment**	**Outcomes**
		**N total**	**N by group**	**Model**	**Gender**	**Age/weight**							
Setkowicz (41)	Poland	33	Exp=15 / Ct=18	Winstar rats	both-preference for males	35 days old/not informed	treadmill and swimming	45 days	2 out of 3 days	20 min	pilocarpine	Convulsive behaviorand EEG	Exercise reduced seizures susceptibility
Rambo (34)	Brazil	48	Be: Exp=8 / Ct=8 EEG: Exp=8 / Ct=8 BA: Exp=8 / Ct=8	Winstar rats	male	Adult/250-300g	swimming	5 weeks	5 days/week	60 min	pentilenetetrazol	Convulsive behavior, EEG, and BA	Exercise attenuated seizures susceptibility and oxidative damage
Reiss (39)	USA	122	Be: Exp=61 / Ct=61 several doses BA: Exp=61 / Ct=61 several doses	Sprague-Dawley rats	male	Adult/150-200g	voluntary wheel running	3 weeks	7days/week		kainic acid	Convulsive behavior and BA	Exercise reduced seizure susceptibility
Souza (35)	Brazil	48	Be: Exp=8 / Ct=8 EEG: Exp=8 / Ct=8 BA: Exp=8 / Ct=8	Winstar rats	male	90 days old/250-300g	swimming	6 weeks	5 days/week	60 min	pentilenetetrazol	Convulsive behavior, EEG, and BA	Exercise reduced seizure susceptibility and EEG spike amplitude
Tutkun (36)	Turkey	14	Exp=7 / Ct=7	Winstar rats	male	Adult/180-200g	swimming	90 days	7 days/week	15-30-60 min	penicillin	EEG	Short-duration exercise decreased the mean frequency and amplitude of epileptiform activity
Gomes da Silva (28)	Brazil	28	Exp=14 / Ct=14	Winstar rats	male	21 days old/ 45-50g	treadmill	39 days	7 days/week	60 min	pilocarpine	Convulsive behavior	Early life exercise may result in the development of more complex neural circuitry capable of tolerating greater brain damage in later life
Kim (37)	Korea	22	Exp=11 / Ct=11	ICR mice	male	Adult/35g	swimming	6 weeks	5 days/week	60 min	kainic acid	Convulsive behavior and BA	Exercise decreased seizure activity and mortality
Kim (38)	Korea	18	Exp=9 / Ct=9	ICR mice	male	Adult/35g	swimming	7 weeks	3 days/week	60 min	kainic acid	Convulsive behavior and BA	Exercise decreased seizure activity and mortality
Hrnčić (52)	Serbia	54	Be: Exp=8/ Ct=8 EEG: Exp=8/ Ct=8 BA: Exp=6/ Ct=6	Winstar rats	male	Adult/180-220g	treadmill	30 days	7 days/week	30 min	homocysteine thiolactone	Convulsive behavior, EEG and BA	Exercise decreased HCT- induced seizure susceptibility
Holmes (40)	USA	36	Be: Exp=11 / Ct=11 EEG: Exp=7 / Ct=7	Sprague-Dawley rats	male	Adult/150-200g	voluntary wheel running	3 weeks	7 days/week		kainic acid	Convulsive behavior and EEG	Exercise reduced seizure severity and hippocampal glutamate release
Kayacan (30)	Turkey	32	Exp=16 / Ct=16	Winstar rats	male	20-24 weeks old/280-350g	treadmill	13 weeks	5 days/week	15-30-60 min	penicillin	EEG	Exercise decreased the frequency of induced epileptiform activity
Kayacan (31)	Turkey	14	Exp=7 / Ct=7	Mongolian gerbils	male	10 weeks old/34-48g	treadmill	8 weeks	7 days/week	30 min	penicillin	EEG	Exercise decreased the spike/wave frequency
Vannucci Campos (42)	Brazil	44	Exp=28 / Ct=16	Winstar rats	female	Adult/220-250	treadmill and voluntary wheel running	6 weeks	5 days/week	30 min (treadmill)	pilocarpine	Convulsive behavior	Voluntary exercise reduced seizure susceptibility
Kayacan (32)	Turkey	16	Exp=8 / Ct=8	Winstar rats	male	20-24 weeks old/280-350g	treadmill	10 weeks	5 days/week	30 min	penicillin	EEG	Exercise decreased spike frequency
Kayacan (32)	Turkey	14	Exp=7 / Ct=7	Winstar rats	male	19-23 weeks old/275-340g	treadmill	10 weeks	5 days/week	30 min	penicillin	EEG	Exercise did not reduce the spike frequency and amplitude

*Ct, control; Exp, experimental; Be, behavior analysis; BA, biochemical analysis; EEG, electrophysiological analysis*.

### Excluded Studies

After reading in full, a total of 10 studies were excluded for the following reasons: (1) exercise program after brain insults or the animals had already had seizures before the physical exercise program (43–46); (2) ascorbic acid in addition to exercise (47); (3) did not evaluate behavioral or electrophysiological manifestations (48); (4) the therapeutic efficacy of regular physical exercise in an animal model with epilepsy and hypertension, since the objective of our to analysis was not to study the exercise in the presence of other comorbidities (49); (5) a book chapter was excluded because it did not report a study model or how it was developed; it described several studies corroborating the exercise benefit (50); (6) no data with specific information were available (51) and (7) acute exercise protocol (52).

### Methodological Quality Assessment

We assessed the quality of each individual study using the 10-item checklist of CAMARADES (Collaborative Approach to Meta-Analysis and Review of Animal Data in Experimental Stroke) (53, 54). The criteria comprise (1) publication in a peer-reviewed journal, (2) statement of control of temperature, (3) randomization to treatment or control, (4) blinded induction of SE (i.e., concealment of treatment group allocation at the time of induction of SE), (5) blinded assessment of outcome, (6) a measure of trainability and inclusion of scale 3 or above animals, (7) adaptation/familiarization to exercise apparatus, (8) sample size calculation, (9) statement of compliance with regulatory requirements, and (10) statement regarding possible conflicts of interest. The median range quality score for the 15 included studies was 5.4 ± 1.14 (range 4–9). All articles were published in peer-reviewed journals. Control of temperature during surgery was documented in 11 of the 15 studies (73.3%), and random allocation to groups was described in 12 of the 15 studies (80%). Allocation concealment was reported in 1 of 15 studies (6.6%), whereas blinded assessment was documented in 4 of 15 studies (26.6%). A measure of trainability and inclusion of scale 3 or above animals was reported in 3 of 15 studies (20%), whereas the use of adaptation and familiarization to exercise apparatus was described in 13 of 15 studies (86.6%). Performing a sample size calculation was not documented in all included studies, a statement of compliance with regulatory requirements was reported in all included studies, and a statement of conflicts of interest was reported in 7 of 15 studies (46.6%) (Table 2).

**Table 2:** The quality of included studies based on the CAMARADES checklist.

**Author**	**Year**	**1**	**2**	**3**	**4**	**5**	**6**	**7**	**8**	**9**	**10**	**Quality score**
Setkowicz	2006	Y	Y	N	N	Y	Y	N	N	Y	N	5
Rambo	2009	Y	Y	Y	N	N	N	Y	N	Y	Y	6
Reiss	2009	Y	Y	Y	N	Y	N	Y	N	Y	N	6
Souza	2009	Y	Y	Y	N	N	N	Y	N	Y	Y	6
Tutkun	2010	Y	Y	N	N	N	N	Y	N	Y	N	4
Gomes da Silva	2011	Y	Y	N	N	N	Y	Y	N	Y	N	5
Hrnčić	2014	Y	Y	Y	N	N	N	Y	N	Y	N	5
Kim	2013	Y	Y	Y	N	N	N	Y	N	Y	N	5
Kim	2014	Y	Y	Y	N	N	N	Y	N	Y	N	5
Holmes	2015	Y	Y	Y	N	Y	N	N	N	Y	Y	6
Kayacan(a)	2016	Y	N	Y	N	N	N	Y	N	Y	Y	5
Kayacan(b)	2016	Y	N	Y	N	N	N	Y	N	Y	Y	5
Vannucci Campos	2016	Y	Y	Y	Y	Y	Y	Y	N	Y	Y	9
Kayacan	2019	Y	N	Y	N	N	N	Y	N	Y	N	4
Kayacan	2020	Y	N	Y	N	N	N	Y	N	Y	Y	5

### Risk of Bias in Included Studies

**Figure 3:**
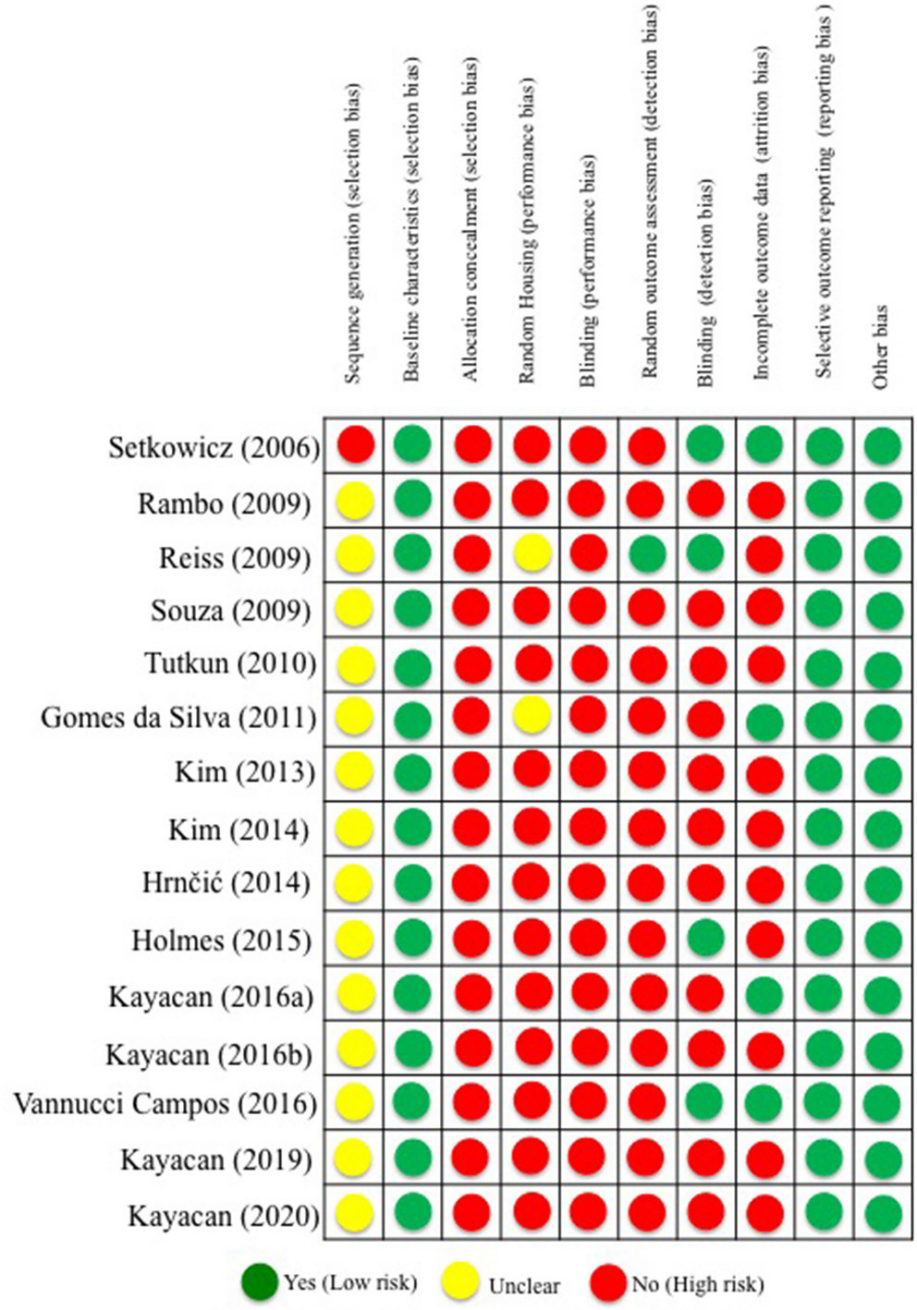
Risk of bias assessment of selected studies.

The assessment of the risks of bias for all included studies can be seen in Figure 3. All studies had a low risk of selection bias considering baseline characteristics and selective outcome reporting. However, regarding selection and performance bias, studies presented a high and unclear risk of bias to sequence generation, allocation concealment, blinding performance, random housing, and blinding. Blinding detection and allocation concealment bias were the criteria of highest declared risk in the studies (100%), followed by the random outcome assessment (93.34%). The data from incomplete results regarding the trend of random attrition, the reports of selective results, the concealment of selection, or any other prejudice presented a high risk of bias in most studies. Selection bias was determined by evaluating how the groups were selected for the study. For this reason, we analyzed if their baseline characteristics were specified and uniform across all subjects, if their selection was determined at random, and if their housing conditions were different. The study by Setkowicz et al. (41) presented a high risk of bias and the other studies were unclear. We determined blinding bias by evaluating if the studies reported randomness in their selection and assessment process, as well as an unbiased appraisal of the results. To be considered to have a low risk of bias, the study must give information as to whether those processes were randomized. We determined the attrition bias by evaluating if the studies reported causes of exclusions when comparing their initial and final n. If there are exclusions, the study must report the causes of exclusion to be considered low risk of bias. We evaluated selective reporting by verifying if the studies fully disclosed their pertinent results, both as graphics and in the text. For a study to be considered of low risk of bias, it must inform their precise data in all forms. Other possible bias sources, such as contamination, design-specific risks, and analysis errors, were evaluated in this category. The lack of them characterizes a low bias risk study (Figure 3).

### Effects of Interventions

From 15 included studies, 13 demonstrated that physical exercise reduced seizure susceptibility induced by different chemoconvulsants such as homocysteine thiolactone (HCT), pentylenetetrazole (PTZ), penicillin, kainite, or pilocarpine (28–32, 34–41) and two studies revealed no significant difference in seizure susceptibility between the exercise and control groups (33, 42), which suggests a potential protective effect of exercise against seizure susceptibility.

#### Behavioral Analysis

Behavioral analysis from the above studies included the latency for the first motor signs, the intensity of motor signs, seizure incidence, latency for reaching SE, number of animals that developed SE, and mortality induced by different chemoconvulsants. Of the studies, 10 (337 animals) analyzed the behavior manifestation following chemoconvulsants.

Of 7 studies reporting latency for the first motors signs or first seizure, four studies showed increased latency (28, 29, 35, 41) and three studies did not find significant alterations following exercise (34, 40, 42).

In relation to the intensity of motor signs, six of ten investigations reported lower intensity following physical exercise (28, 37–41), one registered higher intensity in voluntary but not in forced exercise (42), one reported no significant difference (29), and two did not analyze this parameter (34, 35).

Concerning the number of seizures occurring from chemoconvulsants, one study showed a reduction (29) and eight studies did not analyze the incidence of seizure following exercise (28, 34, 35, 37–39, 41, 42).

Pertaining to SE, the latency for reaching SE was increased in two studies following exercise (41, 42), no significant alterations were observed in one study (28), and in six investigations this parameter was not analyzed (29, 34, 35, 37–39).

In addition, the number of animals that developed SE was analyzed. Six studies showed a reduction of SE development following exercise. Two studies did not find a significant difference between control and exercise groups (28, 42); however, the number of animals that reached SE was not reported in the other studies (29, 37, 38, 41). In four of these studies, SE was described as the severity of seizures, i.e., “5 rating scale for continuous generalized seizures and death within 2 h” (37, 38), rating scale “4 for prolonged severe tonic-clonic convulsions lasting over 20 s—SE frequent repeated episodes of clonic convulsions for an extended period of time—over 5 min” (29) or rating scale 3 for loss of postural tone with general body rigidity (41). Gomes da silva et al. (28) and Vannuci Campos et al. (42) clearly describe “animals that developed SE.” The other three studies that verified the behavior manifestations following chemoconvulsants did not analyze the SE (34, 35, 39).

The mortality rate was investigated in two of the ten studies that explored behavior manifestations following chemoconvulsants (37, 38). In both studies, low mortality was observed following regular physical exercise.

#### Electrophysiological Analysis

Nine studies investigated electrophysiological analysis following chemoconvulsants. A reduction of electrographic seizure activity in exercised animals was found in eight studies (29–32, 34–36, 40) and one did not find significant alterations following exercise (33). Specifically, physical exercise reduced the spike/wave frequency in five studies (29–32, 36) and did not induce a significant alteration spike/wave frequency in another investigation (33). Concerning the spike/wave amplitude, four investigations reported a reduction (31, 34, 35, 40), and six did not find significant alterations (32–36, 40) following exercise.

#### Biochemical Analysis

From the selected studies in this review, 7 evaluated biochemical changes induced by chemoconvulsants following physical exercise (29, 34, 35, 37–40). Among the biochemical variables assessed, five mentioned that exercise prevented oxidative stress (29, 34, 35, 37, 38). One study (39) showed decreased c-fos mRNA autoradiographic density in the hippocampus and increased galanin mRNA optical density in this region and another reported that increased hippocampal glutamate induced by chemoconvulsant was attenuated in exercised rats.

#### Meta-Analysis

**Figure 4:**
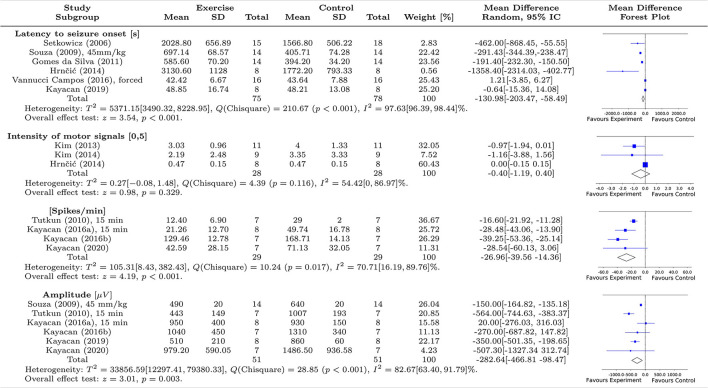
Summary of the meta-analysis on the effect of exercise on seizure susceptibility in animal models. The first column shows the studies included in this analysis, while the subsequent columns display the statistical features, mean and standard deviation (STD), related to experimental and control groups, respectively. The seventh column shows the weights associated with each study, calculated from the variance and number of animals. The eighth column shows the mean effect differences and their respective confidence intervals (CI 95%), assuming a random-effects approach, with between- and within-study heterogeneity given by T^2^ and I^2^. The last column displays the Forest plots associated with each analysis of each epileptogenic characteristic. We evaluated four features associated with the epileptogenic condition; two behavioral features: latency to seizure onset (6 studies) and intensity of motor signals (3 studies); and two electrophysiological features: spike/min (4 studies) and amplitude of spikes (6 studies). Of the behavioral characteristics, only latency to seizure onset showed an overall significant effect (*z* = 3.54, *p* < 0.001) while the two electrophysiological characteristics showed overall statistical significance in favor of exercise to mitigate seizure susceptibility, (spike/min: *z* = 4.19, *p* < 0.001, and amplitude of spikes: *z* = 3.01, *p* = 0.003).

This section comprises a brief explanation about the meta-analysis carried out in this work and how it was applied to the available dataset, including the criteria used to consider or not a sample (which, in this case, is a specific study from the literature) and appropriate types of statistical tests. For the present work, the hypothesis of physical exercise (intervention) being able to reduce or mitigate factors related to the occurrence of epileptic seizures was tested. In total, such factors are four behavioral/electrophysiological variables measured from both experimental (1) and control (2) groups: latency to seizure onset (seconds), the severity of motor signals (measured on a scale from 0 to 5), number of spikes/minute, and amplitude of electrophysiological recordings (μV). Three to six studies were associated with each of these variables, depending on the availability of data and type of experiment conducted. The results were calculated and organized as shown in Figure 4. A more comprehensive discussion about its findings is presented below.

##### Latency to Seizure Onset

For this variable, six studies were included (28, 29, 32, 35, 41, 42). A left-oriented test was adopted but inverting the order of the means $MD_{n}={\bar{x}}_{n,2}-{\bar{x}}_{n,1}$. As discussed in Borenstein et al. (26), the direction of the effect is arbitrary but can be conveniently defined according to its application. Since a longer latency, in terms of epileptiform activity is desired in clinical trials, this notation was adapted to visually match the other ones, which point to the negative direction for a successful intervention. In this case, a global favorable effect linked to the experimental group was statistically significant with *z* = 3.54, *p* < 0.001, where despite the extreme heterogeneity observed between the studies *T*^2^ = 5371.15 [3490.32, 8228.95], the global effect was evidenced by the chi-squared test χ^2^ = 210.67, *p* < 0.0001, and *I*^2^ = 97.63% [96.39, 98.44]%. Considering this extreme heterogeneity between the studies, it indicates the need for further investigation into this variable so that higher stability in the meta-analysis can be attained, and better conclusions can be made.

##### Intensity of Motor Signals

For this variable, three studies were considered. A left-oriented test was adopted, which implies that $MD_{n}={\bar{x}}_{n,1}-{\bar{x}}_{n,2}$ since the expected outcome is negative, that is, lower intensity represents a successful intervention. The main limitation imposed by this variable is due to the lack of a consensual metric scale. Consequently, for comparative analysis to be performed on the same scale, we chose to include only three studies. Besides, all of them contain data represented in terms of median and interquartile ranges. To address this issue, the following approximations were used to convert medians into means and interquartile ranges into standard deviations (55, 56):


(5) $$\begin{array}{r} {\bar{x}}_{n,i}\approx \frac{q_{n,i,1}+m_{n,i}+q_{n,i,3}}{3} \end{array}$$

(6) $$\begin{array}{r} s_{n,j}\approx \frac{q_{n,j,3}-q_{n,j,1}}{1.35} \end{array}$$

where *q*_1_ and *q*_3_ are the first and third quartiles, respectively, is the median value, and, for the intervention and control groups of the study, respectively.

Although the heterogeneity between the studies is not substantial, *T*^2^ = 0.27 [−0.08, 1.48], and *I*^2^ = 54,42% [0, 86.97]%, the global effect was not favorable, with *z* = 0.98, *p* = 0.329. Additionally, all confidence intervals cross the decision threshold on the Forest plot, implying that none of them presented a favorable result toward exercise. The one with the highest weight presented a mean effect located exactly on the threshold (29). This is a situation of difficult quantitative evaluation since the large heterogeneity among studies can hardly be explained due to the low number of studies analyzed. Therefore, we can only evaluate the results descriptively. Therefore, due to a global unfavorable result, and the lack of samples, further investigation into this variable is necessary so that a higher stability in the meta-analysis can be attained, and better conclusions can be made concerning the intensity of motor signals in animal models of epilepsy.

##### Number of Spikes/Minute

We included four studies for this variable, all of which were based on aerobic exercise and convulsant action of penicillin. A left-oriented test was adopted, which implies that $MD_{n}={\bar{x}}_{n,1}-{\bar{x}}_{n,2}$ since the expected outcome is negative (that is, fewer spikes represent a successful intervention). Note that a global significant effect favors the experimental group (animals that were submitted to the exercise program), with *z* = 4.19, *p* < 0.001, and evidenced by the chi-squared test χ^2^ = 10.24, *p* = 0.017. Although one study (33) presented an unfavorable standard deviation in relation to the decision threshold on the Forest plot, there is a predominant tendency in favor of the experimental group (exercise) in all works. However, since the heterogeneity obtained was considerable high between studies, *T*^2^ = 105.31 [8.43, 382.43] and *I*^2^ = 70.71% [16.19, 89.76]%, it becomes hard to infer a final conclusion on this effect.

Most of this high heterogeneity occurs because of the sampling made in the study by Kayacan et al. (33). The high confidence interval presented inthis study (33) is likely due to a lack of significant difference between groups. The weight of the Tutkun et al. study (36) is comparatively higher since the estimations of its mean and confidence intervals are more stable.

Therefore, although this result indicates that exercise programs can significantly decrease the number of spikes/min in animal models of epilepsy, due to the high heterogeneity further investigations are still necessary.

##### Amplitude (μV)

For this variable, we included six studies, three of which presented a significant effect in favor of the experimental group (exercise). A left-oriented test was adopted, which implies that $MD_{n}={\bar{x}}_{n,1}-{\bar{x}}_{n,2}$ since the expected outcome is negative, that is, lower amplitudes represent a successful intervention. However, from the remaining three studies whose confidence interval crossed the decision threshold, two had a favorable mean toward the experimental group. This effect, associated with the work of Souza et al. (35)—which presents good stability in terms of the estimations of mean, confidence intervals, besides a higher sampling –, leads to greater reliability in the analysis, thus assuring a global favorable result toward the experimental group.

Although we observed considerable heterogeneity for this feature between-studies, *T*^2^ = 33856.59 [12297.41, 79380.33] and *I*^2^ = 82.67% [63.40, 91.79] %, the global effect was favorable to the experimental group, with *z* = 3.01, *p* < 0.001, and from the chi-squared test χ^2^ = 28.85, *p* < 0.001. The high heterogeneity might be associated with the high variance of the three studies that did not present a significant effect in relation to the decision threshold on the Forest plot, mainly. And the higher deviation effect found in the Kayacan et al. study (33) is likely due to the lack of statistically significant difference between its groups.

Nevertheless, in summary, this result suggests that exercise can decrease the amplitude of the epileptiform spikes measured from the electrophysiological recordings in animal models of epilepsy.

## Discussion

### Summary of Main Results

The present systematic review and meta-analysis of 15 studies assessed existing evidence of the effects of regular exercise before a precipitating brain insult induced by different animal models of seizures and epilepsy. The included studies indicate that previous exercise training can reduce seizure susceptibility and can protect against the development of epilepsy in animal models, specifically positive changes in behavioral, electrophysiological, and/or biochemical aspects.

#### Effect of Physical Exercise on Behavioral Manifestations of Seizures

Of the 10 studies that evaluated the behavior manifestation, all demonstrated a beneficial effect of exercise in one or more behavioral variables analyzed. Four from eight studies demonstrated that physical exercise delayed the onset of the first seizure induced by chemoconvulsants (28, 29, 35, 41). These findings are in accordance with the first animal study in the literature that evaluated brain susceptibility to seizures using the kindling model of epilepsy (57). In Arida and collaborators' study (57), both acute and chronic exercise retarded the amygdala kindling development. It is important to mention that in general, the rate of kindling has been related to the length of the initial after-discharge, that is, the longer the initial after-discharge, the more rapidly animals develop to stage five of the Racine scale—generalized convulsive seizures (58, 59). Interestingly, the time spent in stage 1 was longer and the after-discharge duration during this stage was shorter in exercise groups compared to the control group. Similar results are observed in other models of seizures or epilepsy. In the pilocarpine (28, 41) or homocysteine model (29), the latency of convulsant-induced symptoms was much longer in trained animals. Of note, although this evidence has not been demonstrated in all studies, most have reported lower intensity of motor signs (28, 37–41). However, our meta-analysis showed an inaccurate effect.

Other important behavioral findings that suggest the beneficial effect of exercise to prevent epilepsy are the latency and the number of animals that developed SE. All studies that analyzed the development of SE (*n* = 105 animals), reported a reduction of SE following exercise. From three studies that analyzed the latency to reach SE, two presented positive outcomes, i.e., the time to SE development was increased in exercised animals (41, 42). This is an important finding because SE has been associated with high mortality and/or brain damage. In addition, high mortality in animal models of epilepsy seems to be in part related to the duration of SE (60). In two selected studies in this review that verified the mortality rate following chemoconvulsants, low mortality was observed following regular physical exercise (37, 38). SE-induced epileptogenesis has been clearly observed in adult animals, and there is substantial experimental evidence for its occurrence in the immature brain (61, 62). In this respect, Novaes Gomes et al. (63) demonstrated that animals subjected to pilocarpine-induced SE in the postnatal period (at P28) and then submitted to a physical exercise program during their adolescent period (between P31 and P90) presented a reduced seizure frequency and beneficial effects on hippocampal plasticity in later stages of life. In line with Gomes da Silva et al. study (28), four weeks of aerobic exercise in hypertensive rats attenuated the development of KA-induced-SE (49). As highlighted by Arida and collaborators (64), suggesting the contribution of physical activity as a potential candidate for stress reduction in epilepsy, one possible mechanism for this effect is that physical stress generated before brain insult induced by SE “can prepare the stress system for new challenges.” However, our meta-analysis showed an inaccurate effect.

#### Effect of Physical Exercise on Electrophysiological Manifestations of Seizures

Several human and animal studies have demonstrated reduced epileptiform discharges on electroencephalography (EEG) induced by exercise. A classic study by Gotze and collaborators in the 1960s reported a reduction in the epileptiform discharge of 30 people with epilepsy submitted to physical effort and hyperventilation (65). Similar findings were observed in subsequent clinical investigations (66–68).

The results of our review are in agreement with the above-mentioned human studies. Eight of nine studies that analyzed electrophysiological parameters showed a reduction of electrographic seizure activity in exercised animals (29–32, 34, 35, 40). From the positive findings, reduced spike/wave frequency and spike/wave amplitude were demonstrated after exercise intervention. Of note, a study reported a reduction of spike/wave frequency in different exercise durations (short, moderate, and long duration) after penicillin injection (30). Positive outcomes were observed in subsequent studies from the same group (31, 32). To reinforce the findings on electrophysiological changes of seizures from this review, an *in vitro* hippocampal electrophysiological investigation demonstrated that trained animals with epilepsy reduced the number of population spikes to potassium and bicuculline extracellular concentrations and partially restored LTP (long term potentiation) impairment observed in animals with epilepsy (69). As mentioned above, these beneficial effects of exercise on electrophysiological manifestation can be particularly prominent for children and adults with epilepsy. Nakken et al. (68) showed that 10 min of exercise until exhaustion diminished the occurrence of EEG alterations in children with epilepsy. Indeed, protocols of intensive exercise until exhaustion have demonstrated benefits in reducing the number of epileptiform discharges on EEG in subjects with temporal lobe epilepsy (12) and juvenile myoclonic epilepsy (13), suggesting that the exhaustive exercise may not be a seizure-inducing factor. The meta-analysis for electrophysiological manifestations showed that previous exercise intervention reduced spike/wave frequency and amplitude, providing a favorable effect of exercise to reduce seizure susceptibility.

#### Effect of Physical Exercise on Biochemical Variables

Seven studies in our review tested the effect of exercise interventions on biochemical changes induced by chemoconvulsant (29, 34, 35, 37–40). Seizures induced by different experimental seizures or epilepsy models increase extracellular glutamate, which consequently contributes to excitotoxic damage. The combination of many changes, such as excitotoxicity induced by glutamate, neuroinflammation, and oxidative stress are neurobiological characteristics of different brain disorders, such as epilepsy (70). To explore underlying mechanisms induced by exercise, the selected studies on this issue revealed some potential pathways of regulation of neural excitability that exercise provides against seizure manifestations. The beneficial effect of exercise to prevent oxidative stress was reported in five of seven studies (29, 34, 35, 37, 38). Oxidative stress plays a critical role in cellular damage and death induced by seizures and the free radicals produced during oxidative stress have been partly recognized to contribute to excitotoxicity (71). Studies in this review clearly demonstrated that physical exercise beneficially altered the anti-oxidative markers after different chemoconvulsants administration such as pentylenetetrazol, kainic acid, and homocysteine.

The role of glutamate to increase seizure excitability has been clearly established (70). One of our selected studies, which measured hippocampal glutamate in real-time using a telemetric, reported that its elevation induced by kainic acid was diminished in exercised animals, indicating a potential protective mechanism that implies a decrease of glutamate release in the hippocampal formation (40). In a posterior study from the same group, Reiss and collaborators (39), tested the role of galanin in regulating neuronal excitability following exercise. Galanin is primarily an inhibitory neurotransmitter, which coexists with norepinephrine in the *locus coeruleus* neurons, and exercise increases galanin gene expression in this region. In their investigation, a previous exercise program decreased seizure-induced c-fos expression in the hippocampus and increased galanin mRNA in *locus coerulrus* (39), suggesting that its protective effect against seizure may be mediated by galanin. Although the selected studies revealed positive effects of exercise interventions on biochemical changes, the present meta-analysis failed to provide any conclusive recommendation considering the limited number of studies on this outcome.

#### Impact of Different Types of Exercise Intervention Before SE Induction

Another important issue to be addressed is which exercise is more adequate to reduce seizure susceptibility induced by chemoconvulsants. Several protocols of physical exercise have been employed to investigate their effects on brain function. The three types of exercise utilized in selected studies, such as voluntary (wheel running), forced (treadmill running), and swimming provided positive results on behavior, electrophysiologic, and biochemical variables. Although in Vannucci Campos and collaborators study (42), no significant behavior alteration from treadmill running was observed, time to SE development was increased in animals submitted to voluntary wheel running. This is the only study that used female animals for this purpose. More exercise investigations in different levels of intensity and duration should be performed to better understand how exercise can impact sex-specific differences in this condition, as clinical studies have already been conducted which show benefits to women with epilepsy (8).

Stress is among the most frequent precipitants of seizures in people with epilepsy, especially emotional stress (72). Exercise can be characterized as physical stress and the scientific literature has reported that physical stress, that is, physical exercise, can induce beneficial effects in both animal models of chronic epilepsy and people with epilepsy (4, 73) We have to bear in mind that activation of this positive stress response may not occur as a consequence of the type of exercise protocol used but also depend on exercise intensities, time of training, rat strains, and sex (74). All exercise interventions analyzed in the selected studies applied exercise of moderate intensity, although duration and session duration varied among studies.

In sum, the efficacy of physical exercise programs for reducing seizure susceptibility in this review was demonstrated in the three exercise models. Of note, there are no animal data in the literature showing the influence of strength-based exercise in this condition. To our knowledge, only two studies using a strength-based exercise program have analyzed its impact on chronic epilepsy induced by pilocarpine. Positively, the strength-based exercise protocol resulted in reducing the seizure frequency in animals with epilepsy (75, 76).

### Study Limitations

The results of the present systematic review and meta-analysis of data from animal studies should be considered within the context of its limitations. As already mentioned, sex-specific differences cannot be entirely ruled out; almost all of the included works have used adult male rats, whereas just one study used females. Although positive findings were observed in studies using exercise interventions before seizure induction on some biochemical profiles, more studies are required to explore the influence of exercise on the brain morphological and neuroinflammatory changes. Although the selected studies have demonstrated the positive impact of exercise on seizure susceptibility in five models of chemoconvulsant rodent models (pilocarpine, kainic acid, pentylenetetrazol, penicillin, and homocysteine), other animal models such as absence seizure models and electroshock-induced seizures might also be investigated for more effective conclusions. As no single model has been validated to study drug-resistant seizures, but rather a number of such models, the same should be employed for analyzing the exercise influence in seizure susceptibility. Thus, from all variables studied, we included only four behavioral/electrophysiological variables in our meta-analysis and three to six studies selected for this purpose. It is important to point out that most of the included studies presented a high risk of bias and poor methodological quality (see Table 2, Figure 3). The high heterogeneity in our meta-analysis is attributable to some factors, including the number of animals considered in each study, different animal models of seizure or epilepsy, different doses of convulsants, and the limited number of studies. The high heterogeneity found in some of our analyses, mainly related to *latency to seizure onset* and *spike/min* features, prevented a robust quantitative evaluation and precise interpretations. We decided to maintain the quantifications, but we stress the importance of further analyses with more similar studies (77).

## Conclusion and Future Directions

It is worth mentioning that, although investigations in humans have been performed to assess the positive effect of exercise on epilepsy, much of the understanding of how previous physical exercise can reduce seizure susceptibility or prevent epilepsy, derives from animal studies. It should keep in mind whether these findings in animal models can be translated to the clinical condition. Indeed, the possible preventive influence of regular exercise to reduce epilepsy incidence in humans is still unclear. To our knowledge, only two studies have investigated this issue. In the first study, a large and population-based cohort, consisting of about 1.2 million men, over a long observation period (up to 40 years) by a Swedish group demonstrated that low cardiovascular fitness assessed at age 18 was associated with an increased risk of presenting epilepsy in later life (14). More recently, another Swedish research group (15) observed that the incidence of epilepsy over 20 years in 197.685 participants of a long-distance Swedish cross-country ski race was up to 40–50% lower before retirement than their match controls. Also, strength or resistance exercise programs have not been explored to identify their impact on seizure susceptibility. It is worth mentioning that people usually include both aerobic and strength training in their physical training routine. In the epileptic condition, the side-effects of some antiseizure medicines can reduce bone density (78), and regular strength-training exercise can revert or reduce these harmful effects. Of note, a recent study with people with epilepsy has demonstrated that a combined aerobic and strength exercise program promoted beneficial effects on cognition (7). Although information concerning these beneficial effects has been observed after epilepsy has been established and considering the positive neuroplastic changes of exercise in animal (63, 79) and human studies (80, 81), previous strength exercise might also exert positive effects before brain insult.

In conclusion, studies selected in this systematic review and meta-analysis indicate that previous physical exercise program reduces seizure susceptibility induced by the administration of different chemoconvulsants. These experimental data suggest an association between previous regular exercise and resilience to developing epilepsy.

## Data Availability Statement

The original contributions presented in the study are included in the article/Supplementary Material, further inquiries can be directed to the corresponding author/s.

## Author Contributions

RA and LT-M created the study design and conceptualized the work. AP, AG, and MR collected the data. JF and JB participated in data acquisition and analysis. RA, LT-M, and RG wrote the manuscript. All authors reviewed and approved the final version of the manuscript.

## Funding

This work was supported by Coordenação de Aperfeiçoamento de Pessoal de Nível Superior—Brasil (CAPES-PRINT # 88881.310490/2018-01); (CAPES # 88887.512294/2020-00); Conselho Nacional de Desenvolvimento Científico e Tecnológico (CNPq # 301732/2018-3; 408676/2018-3) and CAPES - Finance Code 001.

## Conflict of Interest

The authors declare that the research was conducted in the absence of any commercial or financial relationships that could be construed as a potential conflict of interest.

## Publisher's Note

All claims expressed in this article are solely those of the authors and do not necessarily represent those of their affiliated organizations, or those of the publisher, the editors and the reviewers. Any product that may be evaluated in this article, or claim that may be made by its manufacturer, is not guaranteed or endorsed by the publisher.
